# First Pediatric Oncology Unit in India at the Cancer Institute (WIA), Chennai

**DOI:** 10.4103/0971-5851.73604

**Published:** 2010

**Authors:** V. Shanta

**Affiliations:** *Cancer Institute (W.I.A), Adyar, Chennai, India*

Being a Charitable Voluntary Institution, majority of our patients belong to the lower socio economic groups and are housed in a general ward. It was emotionally traumatizing to have innocent children, who hardly know what they are suffering from, mixed with a crowd of adults with advanced cancers of the oral cavity, breast and others. We felt that it was imperative to provide children with cancer an environment of their own where they can play around and be blissfully unaware of their disease.

The first pediatric oncology unit in the country was inaugurated in 1960 by Smt. Indira Gandhi, who was the President of the Indian National Congress then. By mid 70s, the success stories in pediatric therapeutic oncology were becoming evident globally. We faced the problem of poor survival in pediatric leukemias despite following the MRC protocols.

It was in our search to enhance therapeutic results in pediatric leukemias that started the collaborative protocol with the Pediatric division of NCI and Dr. Magrath, which started initially with the Cancer Institute (WIA), Chennai and then extended to many other centers in the country. It raised the DFS in acute pediatric ALL from a dismal 20% to over 60%. The collaboration also made possible many research projects like the impact of socio economic class in leukemia sub type and others.

Our ethos in pediatric care has been “*They shall always have a Tomorrow*”









